# Development and evaluation of the psychometric properties of a digital questionnaire for the evaluation of perinatal psychosocial needs

**DOI:** 10.1186/s12884-023-06050-1

**Published:** 2023-10-17

**Authors:** Paola Bully, Isabel Artieta-Pinedo, Carmen Paz-Pascual, Arturo García-Álvarez, Ema-Q Group, Sonia Alvarez, Pilar Amorrortu, Mónica Blas, Inés Cabeza, Itziar Estalella, Ana Cristina Fernández, Gloria Gutiérrez de Terán-Moreno, Kata Legarra, Gorane Lozano, Amaia Maquibar, David Moreno-López, Mª Jesús Mulas, Covadonga Pérez, Angela Rodríguez, Mercedes Sáenz de Santamaría, Jesús Sánchez, Mª José Trincado, Gema Villanueva, Maite Espinosa

**Affiliations:** 1 Methodological and Statistical Consultant, C/ Barrio La Sota, 48190 Sopuerta, Spain; 2 Osakidetza-Basque Health Service, Biocruces-Bizkaia Health Research Institute, C/ Edificio Biocruces 3. Plaza De Cruces, 48903 Barakaldo, Spain; 3 Primary Care Midwife Zuazo Health Centre, OSI BARAKALDO-SESTAO-OSAKIDETZA, C/ Lurkizaga Kalea, S/N, 48902 Barakaldo, Spain; 4 Associate Professor of the School of Nursing, University of the Basque Country, C/ Barrio Sarriena S/N, 48940 Leioa, Spain; 5 Primary Care Midwife Markonzaga Health Centre, OSI BARAKALDO-SESTAO-OSAKIDETZA, C/ Antonio Trueba Kalea, 17, 48910 Sestao, Spain; 6 Lecturer in the Midwifery Training Unit of the Basque Country, Hospital de Basurto-OSAKIDETZA, C/ Montevideo Etorbidea 18, 48013 Bilbao, Spain

**Keywords:** Pregnancy, Psychosocial needs, Digital questionnaire, Psychometric properties

## Abstract

**Background:**

If the purpose of maternal education is for women to take control of their own health and that of their family in the process, it is essential to have a simple instrument that allows them to self-assess, globally, how prepared they are to face future childbirth and maternity. As there is nothing similar in our area, the objective of this study was to design a complete, specific measurement questionnaire, with good metric quality and in digital format, for the assessment of perinatal psychosocial needs.

**Methods:**

A cross-sectional study was carried out, to evaluate the psychometric properties of a digital measurement questionnaire. The questionnaire was developed in 4 steps following the recommendations of the International Test Commission. The participants were 263 pregnant women who were recruited in primary health care appointments in the Basque Healthcare Service (Osakidetza); they completed the newly created questionnaire and all the test selected as gold standard. Their mean age was 33.55 (SD = 4.73). The analysis of the psychometric characteristics was based on mixed expert judgment procedures (focus group of healthcare professionals, item assessment questionnaire and interviews with users) and quantitative procedures (EFA, CFA, association with the gold standard and classification agreement index, ordinal alpha and McDonald's omega).

**Results:**

The final version of the questionnaire was made up of 55 items that evaluate 8 aspects related to perinatal psychosocial well-being (anxious-depressive symptoms, pregnancy acceptance, partner support, coping, internal locus of control, childbirth self-efficacy, perception of childbirth as a medicalized event, and fear of childbirth). Various tests were made of the validity and reliability of the scores, providing metric guarantees for their use in our context.

**Conclusions:**

The use of this complete, quick-to-use tool with good psychometric properties will allow pregnant women to take stock of their situation, assess whether they have the necessary resources in the psychological and social sphere, and work together with midwives and other health professionals in the areas that are lacking.

## Introduction

Traditionally, over time and across cultures, pregnant women have been surrounded by knowledgeable women, family members and close friends who supported the transition to motherhood. Although pregnancy and childbirth entailed more risks than today, women were considered “experts” in their own pregnancy and in the care of their babies [1]. However, since the emergence of obstetrics and the explosion of new biomedical technologies that have facilitated a preventive approach, pregnancy and childbirth have begun to be treated as risk events, which should be controlled and managed by “experts” [2]. Women have lost their status as an expert in their own pregnancies and have lost psychological confidence. Against this background, Maternal Education (ME) has emerged as a resource for guidance and support for women [3]. ME today is a complex health intervention, which should not only be aimed at teaching a combination of skills and knowledge, but also at providing comprehensive support to foster self-management and self-care during pregnancy, childbirth, puerperium and parenting [4]. Women today demand continuous, accessible, rigorous and personalised ME [5, 6].

In accordance with these requirements, our team has designed an e-Health tool to support Maternal Education, through a collaborative research process [5–8]. The resulting tool has been conceived as a complement to ME, with resources that facilitate its accessibility, continuity and adaptation to the needs of each woman. This tool, EMAehealth, includes: (1) an information area, which is systematically updated according to clinical practice guidelines, (2) a communication area, which allows women to interact with other users and the midwife, (3) a self-management health area, which includes tools for women to self-assess their own health needs, as a basis for informed and/or shared decision-making, and (4) a clinical data area, with access to their own clinical records [7].

In the search for valid, reliable instruments for self-assessment of health needs during pregnancy, we have found that multidimensional instruments aimed at self-assessment of psychosocial needs are practically non-existent. This is despite a growing number of studies reporting an association between mothers' prenatal emotional status and support and their children's socio-emotional and developmental outcomes [9–12], as well as women's emotional health in the postpartum period, or the likelihood of breastfeeding [13]. Poor maternal mental health has also been shown to be more strongly associated with smoking, alcohol and other substance abuse in pregnancy than other known risk factors, including socioeconomic status or maternal age [14].

Most of the instruments found in the literature within the scope of psychological and social needs are aimed at the specific evaluation of one clinical entity such as depression [15, 16], anxiety [17–20], stress [21, 22] and fear of childbirth [23, 24]. A few evaluate social support [25–27] or that of the partner [28, 29], as well as the coping strategies used by the woman to deal with the stress that the imminent childbirth may cause [30–33], the locus of control style [34] or childbirth self-efficacy [35]. In addition, many of them lack psychometric analyses carried out with a Spanish sample.

However, if the objective is for a woman to take control of her own health and that of her family, it is essential that there should be a simple instrument that allows the woman to self-assess how prepared she is to face the future birth and maternity in a global way. In this respect, a valid, reliable tool, adapted to our cultural and social situation, will allow her to take stock of her situation, assess whether she has the necessary psychological and social resources, and work on the areas that turn out to be more deficient or request information, help or advice; in short, to move from having a role of passive recipient of health services to taking an active role, making informed and well-founded decisions [36].

Therefore, given the scarcity of complete, specific questionnaires with proven metric qualities for assessing perinatal psychosocial needs, the objective of this study was to design an appropriate measurement questionnaire for our context in digital format which meets these requirements.

## Method

### Design

This study is part of a broader piece of research in which the perceptions and needs of women during pregnancy, childbirth and postpartum have been analysed, as well as the resources available to them to adapt to each moment of the process. The protocol developed is now available for consultation [37].

This is a cross-sectional study to evaluate the metric characteristics of a digital tool for detecting needs during pregnancy which was carried out between January 2019 and December 2020 in the Basque Public Health Service (Osakidetza). It is a service that provides healthcare to a population of just over two million inhabitants and currently has 7 hospitals where deliveries are performed. Each hospital coordinates with a set of primary health care centres to monitor pregnancy, labour and postpartum.

### Procedure

The questionnaire for Detection of Needs during Pregnancy was created in four steps:

- 1) Focused review of the existing scientific literature for the initial development of the questionnaire.
  Two bibliographical reviews were carried out in the following English and Spanish databases: PubMed, Web of Science, Embase, CINAHL, PsychLIT, PsycINFO, PsicoDoc, IBECS, Cochrane library plus and Google Scholar.
  In the first review, articles related to women’s needs during pregnancy were searched for in order to identify relevant constructs and define them. This work was reviewed by a group of midwives belonging to the research team, and they evaluated its suitability for the context where the study is being carried out.
  In the second review, instruments were searched for which measured the previously identified constructs, with a double purpose: 1) to operationalise them by generating a pool of items and 2) to choose the gold standard. To assess the quality of the metric properties of the existing tools, the “consensus-based standards for the selection of health measurement instruments (COSMIN)” checklist was used. This checklist describes the validity [content validity, construct validity (structural validity, hypothesis testing, cross-cultural validity), and criterion validity], reliability (internal consistency, test–retest reliability, measurement error), and responsiveness of a questionnaire [38].
- 2) Review of the constructs and items by a committee of experts.
  For the initial elaboration of the questionnaire, a multidisciplinary team was formed, with 7 primary care midwives, 5 hospital care midwives, 2 paediatricians, 3 psychologists, 3 methodologists (1 psychometrist and 2 primary health care researchers) and 2 pregnant women. In the content validity evaluation, 6 experts participated to whom was sent a cover letter and the questionnaire explaining why were invited to participate, along with clear and concise instructions on how to rate each item. The purpose of the tool to be developed and the definition of the aspects to be evaluated were explained in writing, in order to avoid bias. They experts individually evaluated each of the 128 items in the initial pool with respect to (1) the relevance of each question in the tool (how important the question is) to a positive experience of delivery; (2) the clarity of each question (how clear the wording is); (3) the essentiality of each question (how necessary the question); (4) adequacy for the population to which it is addressed; (5) relevance of the response scale; giving them a score from 0 to 10 in each aspect. In order to calculate the index of content validity (CVI) and the content validity ratio (CVR), scores of 9 or 10 were considered adequate (very relevant or clear or essential). Item-CVI (I-CVI) is the most widely reported approach for content validity in instrument development. I-CVI is computed as the number of experts giving a rating of “very relevant” for each item divided by the total number of experts. Values range from 0 to 1 where I-CVI > 0.79, the item is relevant, between 0.70 and 0.79, the item needs revisions, and if the value is below 0.70 the item is eliminated. The second type of empirical analysis was CVR, which measures the essentiality of an item. CVR varies between 1 and − 1, and a higher score indicates greater agreement among panel members. The formula for the CVR is CVR = (Ne – N/2)/(N/2), where Ne is the number of experts indicating an item as “essential” and N is the total number of experts. The experts could also report (6) recommendations for improvement of each question and (7) if they consider it necessary to add any question not previously collected. The resulting questionnaire was piloted with a sample of 12 women who reported on their perception of the relevance, adequacy and clarity of each of the items.
- 3) Preliminary analysis of the properties of the instrument.
  In this phase, the web layout of the pilot questionnaire and the gold standards selected for each construct were carried out, and administered to a sample of 100 pregnant women.
  The women were recruited by their midwives at a low-risk pregnancy check-up or through peer group information. They were offered the possibility of receiving the link to a questionnaire in digital format. They were also encouraged to share the link with other women in the same situation. All pregnant women over 18 years of age, fluent in Spanish to understand and respond to the questions presented, could be included. When the woman accessed the link, she received information about the characteristics of the study, the type of use that would be made of the data (for research purposes only) and the ability to withdraw from the study at any time without this compromising their standard of care. The questionnaire was only given if the informed consent was accepted.
  Once the information was collected, an analysis was carried out to evaluate the presence and patterns of missing values and outliers. Next, the descriptive statistics of each of the items were calculated (% cases that are chosen in each option, mean, standard deviation, asymmetry and kurtosis) and compliance or not with the basic assumptions underlying the general linear model (GLM). For the analysis of the internal structure, decision-making techniques were applied regarding the optimal number of factors to be extracted within each construct, and exploratory factor analyses (EFA), using the polychoric correlation matrices, were performed. The internal consistency (ordinal alpha) of each dimension was also calculated, as well as how much this indicator would vary if each item were removed. If items had low saturations in their factor of belonging, and their removal increased the internal consistency of the dimensions, they were eliminated.
- 4) Administration and analysis of the metric properties of the final version
  The findings in the pilot test (high communalities, no cross-loadings, strong primary loadings per factor, the high number of indicators per factor and the absence of missing values), and the moderate length of the questionnaire (62items), suggest that a size sample greater than 200 offers adequate statistical power for the CFA of data [39, 40]. In addition, the possible effect of other variables was considered, such as age, parity, nationality (Spanish/immigrant), educational level (low/medium/high), work outside the home (yes/no) and the presence of certain previous risk factors (such as obesity, negative obstetric history), to guarantee the representativeness of the sample. Following the same procedure as in the pilot test for capturing and collecting information, the enrolled health professionals (gynaecology specialists and midwives) asked 341 women to take part in the study, while another 80 were included by other health professionals or by informal contact between participants.
  A preliminary analysis of the information gathered was carried out in order to clean-up the data and verify compliance with the basic assumptions of the GLM. Afterwards, the fit of the models resulting from the EFAs of step 3 was tested by means of confirmatory factor analysis (CFA). Given the ordinal nature of the items, the estimation method used was diagonal weighted least squares (DWLS) on the polychoric correlation matrix. The evaluation of fit of the model to the data was based on the value of the Chi-square/df ratio, together with information provided by the comparative fit index (CFI), the root mean square error of approximation (RMSEA) and its standardization (SRMR). Models with Chi-square/df ratio values ​​less than 5, equal to or greater than 0.90 in CFI, and equal to or less than 0.08 in RMSEA and 0.10 in SRMR were considered acceptable, models with values equal to or greater than 0.95 in CFI, and equal to or less than 0.05 in RMSEA and 0.08 in SRMR were considered good [41, 42]. The pattern of associations with other variables to obtain evidence of external convergence was analysed using the coefficient of Spearman's correlation (r_s_). The degree of agreement in the classification as a normal or problematic score was calculated using the kappa index of agreement (K) with the classification made with the gold standard questionnaires. Finally, the analysis of the internal consistency of the dimensions was carried out using the coefficients ordinal alpha (ordinal α) and McDonald's omega (ω). The statistical program R (v.4.0.2) was used.

## Results

**Fig. 1:**
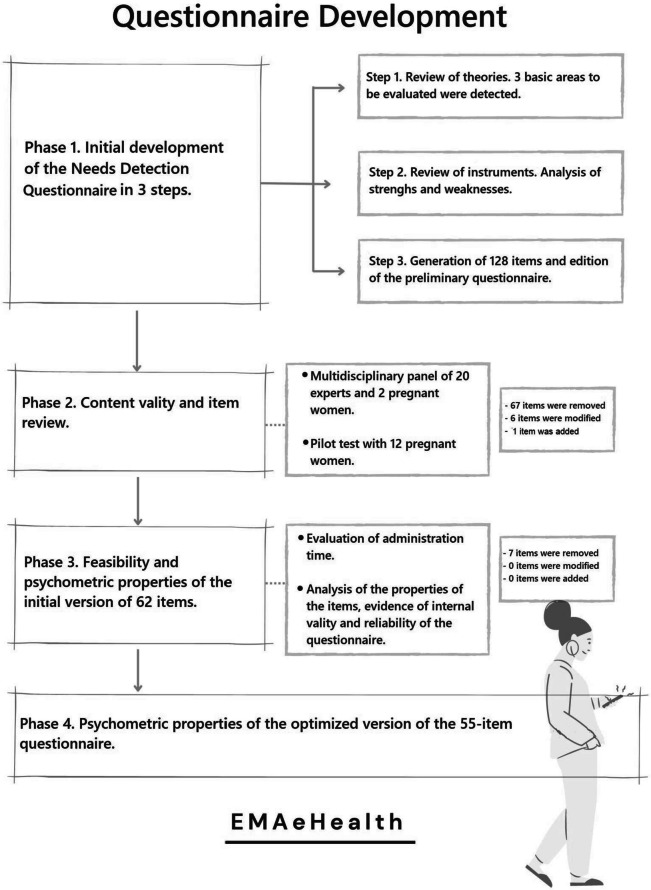
Flowchart of the development process and analysis of the metric properties of the questionnaire

The main results of the four phases which were completed before reaching the optimized version of the questionnaire are described below (see Fig. 1 for a summary).

### Phase 1. Review focused on the existing scientific literature for the initial creation of the questionnaire

After the first review, it was established that the variables that determine the experience of childbirth could be classified into three groups: A) Personal characteristics of the woman (weeks of gestation, physical state, psychological state (screening for anxious-depressive symptoms) and previous or vicarious personal experiences) [43], b) Preferences/desires in relation to the childbirth process [44], and c) Psychological variables and beliefs regarding childbirth, including: (Coping skills/strategies; Need for control/locus of control/beliefs about responsibility (personal or professional) [45]; Self-confidence for the birth [46]; Beliefs about the danger of childbirth [47]; and Fear of the birth [48]). This focussed the objective of the study on the creation of a perinatal psychosocial needs assessment tool.

The second review centred on the search for tools for the evaluation of these psychosocial aspects. As a result of this process, it was concluded that there was a need to create a new, complete, updated tool that is appropriate to our health and digital context.

Table 1 presents the dimensions that were established as most relevant, the questionnaires that would be used as gold standards and the evolution of the number of items remaining to operationalize each dimension.

**Table 1:** Development of the psychosocial needs self-assessment scale during pregnancy. Evolution of the number and distribution of the items

		**Nº Items**				
**Dimensions**	**Gold Standard**	**Initial**	**After experts’ opinion**	**After pilot with 12 women**	**After analysis with 100 women**	**Final questionnaire**
Anxious-depressive symptoms	State-Anxiety Inventory, (STAI) [18] Edinburgh Postnatal Depression Scale (EPDS) [15]	4	4	5	5	5
Acceptance of pregnancy	Prenatal Self Evaluation Questionnaire (PSEQ) [49]: Acceptance	7	3	3	3	3
Partner support	Prenatal Self Evaluation Questionnaire (PSEQ) [49]: Partner support	10	6	6	6	6
Coping skills for childbirth	Revised Prenatal coping Inventory (NuPCI)” [30]	32	13	13	12	12
Locus of control	Multidimensional Locus of Control [34]	24	10	9	5	5
Perceived self-efficacy for the birth	Child Birth Self Efficacy Inventory (CBSEI) [35]	15	11	11	11	11
Attitude towards the birth	Childbirth Attitudes Questionnaire (CAQ) [50]	23	0	0	0	0
Beliefs about the danger of the birth	Medicalization of Childbirth (ATMC) [51]	11	10	10	8	8
Fear of pain in the birth	Wijma Delivery Expectancy/Experience Questionnaire (W-DEQ) [23, 52]	2	5	5	5	5
**Total**		128	62	62	55	55

### Phase 2. Review of the constructs and items by a committee of experts

After the experts committee’s review, the 66 items that obtained an I-CVI < 0.70 were eliminated, leaving almost all the scales with a lower number of items than initially (see columns 3 and 4 Table 1). The I-CVIs ranged from 0.00 to 1.00. Thirty-six items had an I-CVI = 1.00, twenty-six a score of 0.83, twenty-two a score of 0.67, twenty-one a score of 0.50, sixteen a score of 0.33, five a score of 0.17 and two a score of 0.00. Sixty-two items (48.43%) were marked as relevant. The CVR was generated for each item. Items that were marked not essential had a CVR < 0.99 (this value is based on the total number of experts, *N* = 6). Seventeen (13.2%) items had a CVR of 1.00, thirty-six a score of 0.67, thirty-one a score of 0.33, twenty-two a score of 0.00, sixteen a score of -0.33, six a score of − 0.67 and one a score of − 1.00. Nonessential items can be eliminated, but in this case were not due to the loss of information that this would entail. The complete suppression of the dimension ‘Attitude towards the birth’ is to be noted, as it was considered that its content was already covered by other dimensions. In addition, the content of the dimension ‘Fear of pain in the birth’ was expanded by 3 items to include the influence of previous or vicarious experiences, leaving a total of 64 items. Finally, the content of 6 items was reformulated or qualified to increase their clarity and the number of possible responses to each item was homogenized, providing a Likert scale with five alternatives.

After the administration of this version to the group of 12 women, 1 question was added to the ‘Anxious-depressive symptoms’ dimension in order to cover whether or not difficulties are generated in daily activity, and 3 items were eliminated from the ‘Locus of control’ dimension. Additionally, the research team carried out a second review of the eliminated items in case they considered it necessary to reinstate any, reaffirming the decisions made previously.

### Phase 3: Preliminary analysis of the properties of the instrument

To evaluate the comprehensibility, readability, duration and initial properties of the final questionnaire (which was eventually made up of 48.44% of the initial items), it was formatted and a pilot test was carried out with 100 pregnant women.

The results showed that it was a test that the women found easy to use, as it takes around 15 min to complete. In addition, most of the respondents found it easy to understand and interesting.

Based on the findings in the exploratory and internal consistency factor analyses, it was decided that7 items would be eliminated: the 4 items from the external locus of control sub-dimension, 1 item from the positive coping skills dimension, and 2 from the perception of need for medicalization of childbirth dimension. As a result, the optimised version of the questionnaire was left with 55 items.

### Phase 4: Administration and analysis of the metric properties of the final version

#### Characteristics of the participants

Of the 341 women invited to participate by the research team, 183 (54%) actually did so, to which the 80 women attracted by other means were added. Finally, 263 women with a mean age of 33.55 (SD = 4.73) gave their consent and answered all the questionnaires between weeks 8 and 41 of gestation (28.87; SD = 7.34). The characteristics of the participants are distributed in a similar way as in the population of pregnant women [53] and can be seen in Table 2.

**Table 2:** Characteristics of the participants

	**n (%)**
**Age**	
< 30	43 (16.3)
30–34	111 (42.2)
35–39	88 (33.5)
> = 40	21 (8.0)
**Nationality**	
Spanish	232 (88.2)
Foreign	31 (11.8)
**Educational level**	
Without schooling/Primary	8 (3.0)
Secondary school/Vocational training	21 (8.0)
Sixth form/Further Education	91 (34.6)
University degree	143 (54.4)
**Paid employment**	
Yes	222 (84.4)
No	41 (15.6)
**Week of pregnancy**	
< 28	99 (37.6)
28–37	131 (49.8)
> 37	33 (12.5)
**Parity**	
Primiparous	185 (70.4)
Multiparous	86 (29.6)
**Over 40 when the baby is born**	
Yes	26 (9.9)
No	237(90.1)
**Overweight before the pregnancy**	
Yes	33 (12.5)
No	230 (87.5)
**Chronic diseases**	
Yes	37 (14.1)
No	226 (85.9)

Once checked there was no missing or impossible numbers ​​in the database, we proceeded to make a formal description of each of the items (Arithmetic mean (M) and its 95% confidence interval (95% CI), standard deviation (SD), asymmetry index (AI) and Kurtosis index (Ku.)), and evaluate the internal structure, reliability and convergent validity of the questionnaire.

As can be derived from the CFA results for the scale as a whole (55 items, so the factors being specified as the sub-scale items and run within a correlated measurement model), the overall fit of the questionnaire is good (χ^2^ = 2,610.09, df = 1229, *p* < 0.001, χ^2^/df = 2.12, CFI = 0.95, TLI = 0.95, RMSEA (90% CI) = 0.06 (0.06 -0.07), SRMR = 0.08). However, the scale-by-scale analyses are presented below since we are interested that each of them separately also shows good fit so that they can be used in isolation.

#### Anxious-depressive symptoms

This is a one-dimensional scale (χ^2^ = 7.12, df = 4, *p* = 0.129, χ^2^/df = 1.778, CFI = 0.99, TLI = 0.99, RMSEA (90% CI) = 0.05 (0.00 -0.11), SRMR = 0.05) made up of 5 items (see Table 3), with high internal consistency (ordinal α = 0.74; ω = 0.78), high association with the scores obtained in the gold standard (r_s_ = . 70 with the STAI and r_s_ = 0.61 with the EPDS) and a good agreement index in the classification of women as being at risk of suffering from anxiety (K = 0.58) and/or depressive problems (K = 0.33).

**Table 3:** Characteristics of the items that measure anxiety-depressive symptoms

	Min–Max	M	LL	UL	SD	AI	Ku	^λij^
I have felt nervous	0–4	1.78	1.68	1.88	0.85	-0.49	-0.09	.50
I have felt that I could not stop or control my worries	0–4	1.37	1.25	1.48	0.96	0.13	-0.69	.57
I have had little desire to do things	0–4	1.67	1.55	1.78	0.95	-0.11	-0.49	.64
I have felt down, depressed or hopeless	0–4	0.99	0.87	1.11	0.98	0.58	-0.69	.84
I have had difficulty performing normal activities	0–1	0.19	0.15	0.24	0.40	1.56	0.43	.67

^λ^^ij^saturation or weight of the item in the factor

#### Acceptance of the pregnancy

This is also a one-dimensional scale (χ^2^ = 8.76, df = 2, *p* = 0.003, χ^2^/df = 4.38, CFI = 0.92, TLI = 0.91, RMSEA (90%CI) = 0.11 (0.08-. 15), SRMR = 0.06) made up of 3 items (see Table 4), with high internal consistency (ordinal α = 0.76; ω = 0.69), high association with the scores obtained in the gold standard (r_s_ = 0.40) and an acceptable agreement index according to the classification made with the gold standard (K = 0.30).

**Table 4:** Characteristics of the items that measure Acceptance of pregnancy

	Min–Max	M	LL	UL	SD	AI	Ku	λ_ij_
I am very happy to be pregnant	0–4	3.70	3.61	3.78	0.72	-3.00	10.31	.60
This is a planned pregnancy	0–4	3.41	3.29	3.54	1.05	-1.81	2.33	.66
It was difficult for me to accept this pregnancy	0–4	0.44	0.34	0.55	0.90	2.27	4.62	.85

#### Perception of support from the partner

This is a scale made up of 6 items (χ^2^ = 8.23, df = 9, *p* = 0.512χ^2^/gl = 0.91, CFI = 0.99, TLI = 0.99, RMSEA (90% CI) = 0.01 (0.00-0.06), SRMR = 0.02) (Table 5), whose internal consistency (ordinal α = 0.93; ω = 0.90), association (r_s_ = 0.81) and agreement index of the classification with the gold standard were excellent (K = 0.66).

**Table 5:** Characteristics of the items that measure Partner support

	Min–Max	M	LL	UL	SD	AI	Cu	λ_ij_
I know I have the support of my partner when I feel overwhelmed	0–4	3.53	3.44	3.62	0.73	-1.87	4.33	.92
I consider that as a couple we have a good level of communication	0–4	3.45	3.36	3.54	0.76	-1.50	2.42	.91
I believe I will have the support of my partner during childbirth	0–4	3.67	3.59	3.76	0.68	-2.63	8.25	.73
I can count on my partner for the care I need during pregnancy	0–4	3.62	3.54	3.70	0.68	-1.99	4.48	.89
I believe I can count on my partner to help take care of the baby	0–4	3.72	3.65	3.79	0.59	-2.43	7.44	.86
My partner and I talk about the pregnancy whenever I need to	0–4	3.55	3.46	3.65	0.76	-2.02	4.58	.87

#### Coping

This is made up of two subscales, one of 7 items that measures positive coping skills (χ^2^ = 18.98, gl = 12, *p* = 0.089, χ^2^/gl = 2.71, CFI = 0.99, TLI = 0.99, RMSEA (90% CI) = 0.05(0.00-0.85), SRMR = 0.04, ordinal α = 0.75; ω = 0.79,r_s_ = 0.77, K = 0.50) and another of 5 that measures avoidance (χ^2^ = 5.84, df = 5, *p* = 0.322, χ^2^/df = 1.17, CFI = 0.96, TLI = 0.98, RMSEA (90% CI) = 0.05 (0.00-0.11), SRMR = 0.03, ordinalα = 0.71, ω = 0.67,r_s_ = 0.86, K = 0.69) (Table 6), both with excellent metric properties.

**Table 6:** Characteristics of the items that measure Coping

	Min–Max	M	LL	UL	SD	AI	Ku	λ_ij_
I ask doctors or midwives questions about childbirth	0–4	1.69	1.57	1.81	1.00	0.04	-0.26	.54
I think about what things will be like after the baby arrives	0–4	2.95	2.86	3.04	0.75	-0.84	1.89	.54
I plan what I am going to do during childbirth	0–4	1.89	1.76	2.01	1.06	-0.08	-0.59	.78
I spend time with or talk to people who have just had a baby	0–4	1.93	1.81	2.04	0.94	-0.19	-0.37	.50
I imagine how the birth will unfold	0–4	2.03	1.92	2.14	0.92	-0.24	-0.32	.72
I talk to women in my family or friends about what it is like to give birth	0–4	1.93	1.81	2.05	0.98	0.01	-0.30	.65
I look for information in books and on the internet about pregnancy, childbirth, etc	0–4	2.33	2.20	2.46	1.06	-0.40	-0.36	.57
Have you tried not to tell other people about your feelings about the pregnancy?	0–4	0.95	0.84	1.07	0.96	0.62	-0.57	.50
Have you slept to avoid problems?	0–4	0.83	0.72	0.94	0.93	0.81	-0.27	.68
Have you wished, while pregnant, that the birth had already happened?	0–4	1.32	1.17	1.46	1.21	0.50	-0.65	.58
Have you tried to feel better by eating?	0–4	0.94	0.81	1.07	1.08	0.99	0.27	.65
Have you wished you weren't pregnant?	0–4	0.16	0.11	0.22	0.45	2.85	7.53	.45

#### Internal locus of control

This is made up of 5 items (χ^2^ = 13.56, df = 5, *p* = 0.019, χ^2^/gl = 2.71, CFI = 0.98, TLI = 0.97, RMSEA (90% CI) = 0.08 (0.03-0.13), SRMR = 0.05) (Table 7), presents good internal consistency (ordinal α = 0.69; ω = 0.69), high association with the scores in the gold standard (r_s_ = 0.69) and an acceptable agreement index with the classification of the gold standard (K = 0.28).

**Table 7:** Characteristics of the items that measure Locus of Control

	Min–Max	M	LL	UL	SD	AI	Ku	λ_ij_
I am sure my birthing experience will depend on me applying everything I know	0–4	2.21	2.11	2.32	0.88	0.01	-0.04	.54
I am more likely to have the kind of experience I want if I plan the delivery	0–4	1.75	1.63	1.86	0.94	0.09	-0.48	.65
I can largely determine the progress of my labour	0–4	1.97	1.86	2.08	0.91	-0.16	-0.49	.64
Knowing my rights, I will be able to protect my interests during labour	0–4	2.56	2.46	2.66	0.81	-0.29	0.03	.62
My labour will be determined by my own actions	0–4	2.03	1.92	2.14	0.89	-0.13	-0.35	.54

#### Childbirth self-efficacy

This is made up of 11 items (χ^2^ = 151.80, df = 43, *p* < 0.001, χ^2^/df = 3.53, CFI = 0.98, TLI = 0.97, RMSEA (90%CI) = 0.09 (0.08-0.11), SRMR = 0.07) (Table 8), presents very high internal consistency (ordinal α = 0.87; ω = 0.86), high association with gold standard scores (r_s_ = 0.59) and a good agreement index in the classification of at risk of presenting difficulties (K = 0.43).

**Table 8:** Characteristics of the items that measure Self-efficacy for childbirth

	Min–Max	M	LL	UL	SD	AI	Ku	λ_ij_
I believe I canpush enough during labour for my baby to be delivered	0–4	3.13	3.04	3.22	0.72	-0.57	0.55	.81
I will manage well during childbirth, just as with other challenges in my life	0–4	3.05	2.97	3.14	0.70	-0.41	0.49	.84
I think I will be able to stay calm	0–4	2.45	2.35	2.56	0.88	-0.12	-0.44	.75
I will be able to focus on collaborating in the delivery, even if other things are happening around me	0–4	2.91	2.83	3.00	0.72	-0.56	0.91	.73
I think I will feel comfortable giving birth in the presence of the medical team	0–4	2.89	2.79	3.00	0.85	-0.70	0.65	.55
Childbirth will be a satisfactory experience for me	0–4	2.67	2.57	2.77	0.84	-0.12	-0.21	.71
I am fine with the idea that labour might be prolonged over time	0–4	2.03	1.92	2.15	0.97	0.11	-0.57	.59
I think I will be able to detect the moment of labour onset	0–4	2.52	2.42	2.62	0.82	-0.29	-0.06	.50
I think I will be able to get to the hospital at the right time	0–4	2.60	2.51	2.69	0.74	-0.57	0.30	.52
I think my body is perfectly prepared to give birth	0–4	2.97	2.88	3.06	0.76	-0.38	0.12	.63
I am able to bear the pain	0–4	2.44	2.33	2.55	0.88	-0.35	0.10	.64

#### Perception of childbirth as a medicalised process

This is a scale with 8 items (see Table 9) which shows a good fit to a two-dimensional model of related factors (χ^2^ = 37.38, gl = 19, *p* < 0.001, χ^2^/gl = 1.97, CFI = 0.98, TLI = 0.98, RMSEA (90%CI) = 0.06 (0.03-0.09), SRMR = 0.06). The first 4 items evaluate the perception of childbirth as a medical process and present good reliability and convergent validity (ordinal α = 0.79; ω = 0.77, r_s_ = 0.64, K = 0.23) and the other 4 evaluate the perception as a process as natural, also with good metric properties (ordinal α = 0.75; ω = 0.72,r_s_ = -0.44). These aspects correlate inversely (r = -0.34).

**Table 9:** Characteristics in the items that measure the perception of childbirth as a medical process

	Min–Max	M	LL	UL	SD	AI	Ku	λ_ij_
Childbirth requires vigilant medical supervision	0–4	3,21	3,09	3,32	0,97	-1,36	1,57	.69
There are many things that can go wrong during childbirth	0–4	2,79	2,69	2,89	0,82	-0,53	0,34	.68
Childbirth is a medical process		2,25	2,12	2,38	1,04	-0,17	-0,51	.83
Childbirth is a dangerous process		2,10	1,98	2,22	1,00	-0,11	-0,58	.77
Childbirth is an empowering experience	0–4	2,34	2,23	2,46	0,96	-0,04	-0,15	.49
Childbirth is a natural process	0–4	3,36	3,28	3,44	0,66	-0,62	-0,27	.68
A woman's body knows how to react at the time of childbirth	0–4	3,08	3,00	3,17	0,73	-0,43	-0,11	.83
Dilation/labour should be allowed to progress at its own pace	0–4	2,89	2,79	2,99	0,79	-0,33	-0,30	.73

#### Fear of childbirth

This is made up of 5 items (χ^2^ = 4.69, df = 5, *p* = 0.460, χ^2^/gl = 0.94, CFI = 0.99, TLI = 0.99, RMSEA (90%CI) = 0.01 (0.00-0.08), SRMR = 0.04) (Table 10), and presents very high internal consistency (ordinal α = 0.90; ω = 0.87), moderate association with the scores in the gold standard (r_s_ = 0.57) and good fitto the classification of the gold standard (K = 0.40).

**Table 10:** Characteristics of the items that measure Fear of childbirth

	Min–Max	M	LL	UL	SD	AI	Ku	λ_ij_
Level of fear before childbirth	0–10	6.09	5.77	6.41	2.60	-0.35	-0.62	.89
Level of anxiety or nervousness before childbirth	0–10	6.06	5.77	6.36	2.41	-0.42	-0.46	.83
Level of fear of childbirth due to personal bad experiences or those of acquaintances	0–10	4.72	4.39	5.05	2.72	0.07	-0.83	.60
Level of fear of health complications arising from childbirth	0–10	5.56	5.22	5.89	2.75	-0.22	-0.80	.71
Level of fear, at this moment, that the baby will suffer during childbirth	0–10	7.06	6.74	7.38	2.63	-0.70	-0.42	.73

## Discussion

The promotion, prevention, early intervention, and treatment of perinatal psychosocial problems should be a global priority [54, 55]. Given the scarcity of complete, specific evaluation instruments with checked metric quality, a four-phase process has been developed, to produce a digital questionnaire made up of 55 items that evaluate 8 essential aspects for good psychosocial adjustment during pregnancy and successful coping with pregnancy.

Among the issues that are related with adaptation to pregnancy, the following stand out: 1) The screening of anxious-depressive symptoms through 5 items that serve to assess whether the cognitive and emotional state allows the woman to use her mental and social skills; 2) Acceptance of the pregnancy, reflected in 3 items that evaluate the positive response to being pregnant and; 3) The perception of support from the partner, measured with 6 items that include the partner's interest in the woman's needs as a future mother, her adjustment to the new paternal/maternal role, and empathy shown. These three factors have already been highlighted in the literature, some of them interrelated. For example, identifying low acceptance of pregnancy can potentially improve current medical practice by improving early detection of maternal depression [56], which is a good predictor of general mental health and the future bond that the woman will make with the baby [57].

The aspects that can be decisive for the birth process are: 1) The perception of coping skills made up of 12 items that evaluate the cognitive and behavioural strategies that the woman uses to manage the internal or external demands of a difficult situation such as childbirth; 2) The locus of control or perception that the woman has about the causes of what happens in her life, in this case measured with 5 items that evaluate the attribution/internal control over them; 3) Self-efficacy, or the belief a woman has that she possesses the capabilities to perform the necessary actions that allow her to obtain the desired results during the childbirth process, measured with 11 items; 4) The perception of childbirth as medicalised, which is the a priori belief in the risk that childbirth poses to the life of the mother and the baby and the need for it to be carried out in instrumentalised medical environments, with a list of 8 items and; 5) Fear of childbirth, measured with 5 items, understood as suspicion, apprehension or distressing mood disturbance due to a real or imaginary risk or harm, or fear of something happening contrary to what is desired during childbirth.

All the dimensions showed good global fit to the starting theoretical models, with values ​​of the χ^2^/df ratio between 0.91 and 4.38, making them all under 5, the maximum value recommended by the psychometric community. This also occurred with the RMSEA and SRMR indicators, which ranged between 0.01 and 0.09 in the first case and between 0.02 and 0.07 in the second, with 0.10 being the maximum value tolerated. None of the items presented local fit problems, all saturating above 0.4 in their respective dimensions (the majority above 0.60). The associations of the scores obtained in the dimensions with those found in the gold standard were moderate to high, ranging from 0.40 to 0.86, which is considered good evidence of convergent validity. The values ​​found in the kappa agreement indices between the classifications made with the new tool and the gold standard point in the same direction, varying from 0.23 in the case of the perception of childbirth as dangerous and in need of medicalised assistance, to 0.69 in the case of negative childbirth coping skills. In terms of reliability, we can say that internal consistency was very good in all dimensions, with values ​​between 0.69 and 0.93 in ordinal alpha and between 0.69 and 0.90 in McDonald's omega.

It is important to have a global vision of all dimensions when evaluating and intervening. For example, it has been seen that coping can function as a mediating factor between other factors: greater social support decreased the likelihood of depression, not only directly but also through the mediating role of coping styles [58]. The fear of birth could be concurrent with depressive symptoms [59], and can have a negative impact on a woman’s psychological wellbeing during pregnancy and her experience of birth. It has also been associated with adverse obstetric outcomes and postpartum mental health difficulties [60]. Interventions can consist of working on one dimension to achieve an effect on another or others, and we can also evaluate the dimensions, together or separately, to adapt the intervention methodology to each case.

The greatest strength of this study is the use of a participatory approach, which obtains evidence on the validity of its content from the scientific literature and in a consensual and collaborative manner from health experts and users. It also has good psychometric properties, as it is much shorter than the total of the questionnaires used as the gold standard (55 vs. 173 items), in addition to the possibility of evaluating each area in isolation. Among its uses, it makes it easier to collect information from pregnant women without consuming a great deal of time. The instrument systematically and efficiently evaluates aspects of health that may be difficult for the professional to address in the usual consultation time, facilitating care that is more comprehensive. In maternal education sessions, it can be used to adapt the contents to the needs of the group, and also to evaluate if objectives have been achieved. In addition, it makes it easier for the woman to take an active role in finding the resources she needs to attend to her health and that of her baby, once she has self-assessed her needs. Furthermore, in the longer term this type of tool, in digital format, may prove useful for collecting data at community level. This characteristic will facilitate the progressive and continuous adaptation to the needs of different types of populations (immigration, socioeconomic differences, fathers/mothers), increasing the effectiveness of interventions.

## Limitations

The fact that the selection of the sample was not random might increase the representation of more proactive women with a higher educational level. However, measures have been taken to avoid introducing bias: the women were selected by 25 midwives belonging to public health centres located in various population areas, both rural and urban, and of different socioeconomic and social characteristics. In light of the socio-demographic data, it can be considered that the women in our study are representative of the study population.

The length of the scale was very considerable when it was presented together with the gold standard questionnaires, which might reduce the participation of women with less motivation. To remedy this effect, the questionnaire was presented in digital format, accessible by mobile phone, which favoured its use and management in a young population of all social origins, who use smartphones in their daily lives. The questionnaire also allowed it to be completed in stages.

Finally, there is a lack of evidence regarding the stability over time of the scores, but we expect information on this aspect will be obtained in future studies.

## Conclusions

Based on the results found and the changes made to them, it can be stated that the scores that will be obtained from the final version of the questionnaire have the guarantees of validity and reliability required by the international standards for test creation and adaptation [61].

The result of this work aims to respond to the demand for addressing the mental health of women from a global perspective, centred on the individual [55], in a multidimensional way [55, 62].

## Data Availability

The data sets generated and/or analysed during the current study are not yet publicly available as they are still being processed by the research team for further publication, but will be made available from the corresponding author upon reasonable request.

## Abbreviations

CFA: Confirmatory Factor Analysis
EFA: Exploratory Factor Analysis
AI: Asymmetry index
COSMIN: Consensus-based standards for the selection of health measurement instruments
ME: Maternal Education
M: Arithmetic mean
Min: Minimum
Max: Maximum
LL: Lower Median limit
UL: Upper Median Limit
λij: Standardized factorial weight
95% CI: Confidence interval of 95%
K: Cohen’s kappa coefficient
ω: McDonald's Omega
Ordinal α: Ordinal alpha
SD: Standard deviation
Ku: Kurtosis index
DWLS: Diagonally Weighted Least Squares
χ^2^: Chi-squared
DF: Degrees of freedom
CFI: Comparative Fit Index
TLI: Tucker-Lewis Index
RMSEA: Root Mean Square Error of Approximation
SRMR: Standardized Root Mean Square Residual
